# Analysis of miRNA Expression Profiles in Traumatic Brain Injury (TBI) and Their Correlation with Survival and Severity of Injury

**DOI:** 10.3390/ijms25179539

**Published:** 2024-09-02

**Authors:** Francesca Consalvo, Martina Padovano, Matteo Scopetti, Donato Morena, Luigi Cipolloni, Vittorio Fineschi, Alessandro Santurro

**Affiliations:** 1 Department of Medicine, Surgery and Dentistry “Schola Medica Salernitana”, University of Salerno, 84081 Baronissi, Italy; fconsalvo@unisa.it; 2 Department of Anatomical, Histological, Forensic and Orthopaedic Sciences, Sapienza University of Rome, 00161 Rome, Italy; martina.padovano@uniroma1.it (M.P.); matteo.scopetti@uniroma1.it (M.S.); donato.morena@uniroma1.it (D.M.); vittorio.fineschi@uniroma1.it (V.F.); 3 Department of Clinical and Experimental Medicine, University of Foggia, 71100 Foggia, Italy; luigi.cipolloni@unifg.it

**Keywords:** traumatic brain injury, miRNA, molecular pathology, molecular diagnostics, molecular pathology of traumatic injuries, miR-16, miR-21, miR-130a, miR-155, miR-23a

## Abstract

Traumatic brain injury (TBI) is the leading cause of traumatic death worldwide and is a public health problem associated with high mortality and morbidity rates, with a significant socioeconomic burden. The diagnosis of brain injury may be difficult in some cases or may leave diagnostic doubts, especially in mild trauma with insignificant pathological brain changes or in cases where instrumental tests are negative. Therefore, in recent years, an important area of research has been directed towards the study of new biomarkers, such as micro-RNAs (miRNAs), which can assist clinicians in the diagnosis, staging, and prognostic evaluation of TBI, as well as forensic pathologists in the assessment of TBI and in the estimation of additional relevant data, such as survival time. The aim of this study is to investigate the expression profiles (down- and upregulation) of a panel of miRNAs in subjects deceased with TBI in order to assess, verify, and define the role played by non-coding RNA molecules in the different pathophysiological mechanisms of brain damage. This study also aims to correlate the detected expression profiles with survival time, defined as the time elapsed between the traumatic event and death, and with the severity of the trauma. This study was conducted on 40 cases of subjects deceased with TBI (study group) and 10 cases of subjects deceased suddenly from non-traumatic causes (control group). The study group was stratified according to the survival time and the severity of the trauma. The selection of miRNAs to be examined was based on a thorough literature review. Analyses were performed on formalin-fixed, paraffin-embedded (FFPE) brain tissue samples, with a first step of total RNA extraction and a second step of quantification of the selected miRNAs of interest. This study showed higher expression levels in cases compared to controls for miR-16, miR-21, miR-130a, and miR-155. In contrast, lower expression levels were found in cases compared to controls for miR-23a-3p. There were no statistically significant differences in the expression levels between cases and controls for miR-19a. In cases with short survival, the expression levels of miR-16-5p and miR-21-5p were significantly higher. In cases with long survival, miR-21-5p was significantly lower. The expression levels of miR-130a were significantly higher in TBI cases with short and middle survival. In relation to TBI severity, miR-16-5p and miR-21-5p expression levels were significantly higher in the critical–fatal TBI subgroup. Conclusions: This study provides evidence for the potential of the investigated miRNAs as predictive biomarkers to discriminate between TBI cases and controls. These miRNAs could improve the postmortem diagnosis of TBI and also offer the possibility to define the survival time and the severity of the trauma. The analysis of miRNAs could become a key tool in forensic investigations, providing more precise and detailed information on the nature and extent of TBI and helping to define the circumstances of death.

## 1. Introduction

Traumatic brain injury (TBI) is the leading cause of traumatic death worldwide and a major public health problem in Europe, associated with high mortality and morbidity rates and a significant socioeconomic burden [1,2,3]. Worldwide, the annual incidence of head injury is estimated to be between 27 and 69 million new cases, with road traffic accidents being the leading cause [4].

The pathophysiology of TBI is complex and involves a cascade of events that occur at the time of injury and continue for days or weeks after injury [5]. In particular, TBI can be attributed to two different, partly overlapping, pathogenetic mechanisms, which can be divided into primary and secondary brain damage [6,7,8]. Primary brain damage results from direct mechanical damage to brain tissue, which may be caused by acceleration or deceleration forces, rotational forces, or direct impact. The resulting damage may include contusions, lacerations, and diffuse axonal damage [9,10]. Secondary brain damage follows the activation of biochemical cascades of various types (such as free radical generation, neuroinflammatory response, and cell death) that occur after the initial injury and may lead to further brain tissue damage, including hypoxia, ischaemia, inflammation, and oxidative stress [11,12].

The study of TBI is of great forensic relevance. The diagnosis of brain injury may be difficult in some cases or may leave diagnostic doubts, especially in mild trauma with insignificant pathological brain changes or in cases where instrumental tests are negative [13]. Even in the forensic setting, the diagnosis of brain injury may be difficult, as in cases of diffuse axonal injury (DAI) in which macroscopic examination does not reveal obvious brain changes [14,15].

Therefore, in recent years, an important area of research has been directed towards the study of new biomarkers, such as micro-RNAs (miRNAs), which can assist clinicians in the diagnosis, staging, and prognostic evaluation of TBI, as well as forensic pathologists in the assessment of TBI and in the estimation of additional relevant data, such as survival time [16]. They may also represent valuable therapeutic targets for the treatment of TBI [17,18,19,20,21].

In this sense, micro-RNAs (miRNAs) represent potential and promising biomarkers applicable in clinical diagnostics and forensic science. miRNAs are small non-coding RNA molecules involved in the pathophysiology of many diseases and are crucial for neurodevelopment and brain function [22,23]. By regulating gene activity, miRNAs control cellular processes for neuronal damage and repair: differentiation, proliferation, apoptosis, and metabolism. Altered miRNA levels have been reported in several central nervous system (CNS) disease processes [24], including traumatic brain injury [25]. miRNAs have also been proposed as therapeutic targets for TBI, so several studies have been conducted on murine models in order to evaluate their use [20,21]. For example, the use of inhibitors (antagomiR) or mimetics (agomiR) of miRNAs has been tested as an innovative therapeutic strategy to reduce brain damage and improve clinical outcomes in patients with TBI [26,27].

Compared to known TBI protein biomarkers, miRNAs may be preferable due to their specific characteristics [28]. First, their small size is related to greater stability even in highly degraded samples, such as cadaveric samples. Secondly, their high tissue-specific expression gives them greater sensitivity to the pathology under investigation. Furthermore, due to their action at the post-transcriptional level, miRNAs can be detected in the early stages of a disease, long before the effects of downstream protein expression are observed [29]. For these reasons, several papers have been produced to evaluate the different miRNA expression profiles in subjects with TBI compared to controls [30].

The majority of TBI studies have been conducted on cell lines or biological fluid samples such as plasma, serum, cerebrospinal fluid (CSF), and saliva [31,32,33,34,35]. Only a minority of studies have been conducted on tissue samples (brain tissue), mainly from mouse models subjected to controlled cortical impact [36,37,38,39]. In the currently available postmortem studies of TBI, the expression levels of specific miRNAs on formalin-fixed, paraffin-embedded (FFPE) brain tissue samples have been investigated. In these studies, several miRNAs have been identified as dysregulated in brain injury, such as miR-16, miR-21, miR-92, miR-124, miR-138, and miR-144 [40,41]. Numerous other miRNAs have been implicated in the pathophysiology of TBI and are being investigated for their potential role in both clinical–therapeutic and forensic settings. Despite this evidence, further studies are currently required to validate their use.

This study concerns the profile expression of a set of miRNAs, preliminarily selected based on the literature review, evaluated on formalin-fixed, paraffin-embedded (FFPE) brain tissue samples from subjects who died of TBI, stratified based on the survival time and the severity of the injury.

The aim of this study is to investigate the expression profiles (downregulation and upregulation) of a set of miRNAs in subjects deceased as a result of TBI in order to assess, verify, and define the role of non-coding RNA molecules in different pathophysiological mechanisms of brain injury. This study also aims to correlate the detected expression profiles with survival time, defined as the time elapsed between the traumatic event and death, and with the severity of the trauma.

## 2. Results

### 2.1. Study Group Characteristics

Forty subjects deceased as a result of traumatic brain injury (TBI) were selected: thirty-one (77.50%) were male and nine (22.50%) were female. The mean age of the subjects included in the study group was 45.05 ± 19.17 SD (median 53 years, age min 12, max 65). In particular, 13 subjects were younger than 35 years old, and 27 were older than 35 years old.

The most common cause of death in the study group was major trauma (55%). The injury mechanisms leading to the head injury were a road traffic accident in twenty-five cases (62.5%), a fall from standing in seven cases (17.5%), a fall from a height in four cases (10%), a train accident in three cases (7.5%), and blunt trauma in one case (2.5%). Road traffic accidents were thus the most common mechanism of head injury, accounting for 42.5% of the study group. The most common type of brain injury was subarachnoid haemorrhage (SAH), which was present in 24 of the subjects (60%). In all selected cases, the postmortem interval (PMI) was less than 6 days, with a mean of 80.275 ± 19.599 h (median 76, PMI min 52, PMI max 123). In addition, of the selected subjects, six had undergone surgical treatment by decompressive craniotomy (Table 1).

### 2.2. Control Group Characteristics

Ten subjects who died of natural causes, with a cardiovascular genesis, were selected according to the inclusion and exclusion criteria (Table 2). Six (60%) were male and four (40%) were female, and the mean age of the subjects included in the study group was 53.2 ± 10.97 SD (median 55.5 years, age min 24, max 65).

### 2.3. Subgroup Characteristics

**Figure 1:**
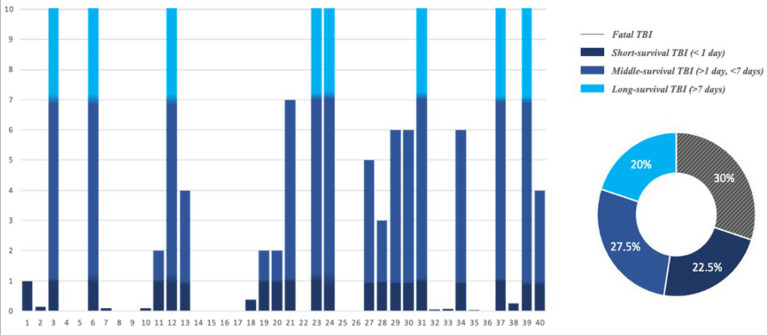
Distribution of study group by survival time expressed in days.

The study group was selected by defining four subgroups consisting of cases distributed according to survival time (Figure 1). Specifically, the four subgroups consisted of twelve cases of fatal TBI (no survival), nine cases of TBI with short survival (survival < 1 day), ten cases of TBI with middle survival (survival >1 and <7 days), and nine cases of TBI with long survival (survival > 7 days).

**Figure 2:**
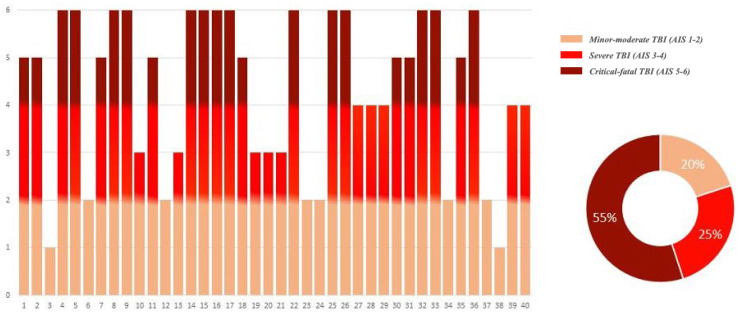
Distribution of study group by severity of trauma, as defined by AIS score parameters.

The study group was stratified in parallel according to the severity of the trauma, which was assessed using criteria derived from the Abbreviated Injury Scale (AIS) score (Figure 2). Specifically, three subgroups were defined, consisting of eight cases of mild–moderate TBI (AIS 1-2), ten cases of severe TBI (AIS 3-4), and twenty-two cases of critical–fatal TBI (AIS 5-6).

### 2.4. miRNA Expression Profiles in Cases and Controls

Complex data analysis revealed expression levels with statistically significant differences—between cases and controls—for hsa-miR-16-5p, hsa-miR-21-5p, hsa-miR-23a-3p, hsa-miR-130a-3p, and hsa-miR-155-5p.

**Figure 3:**
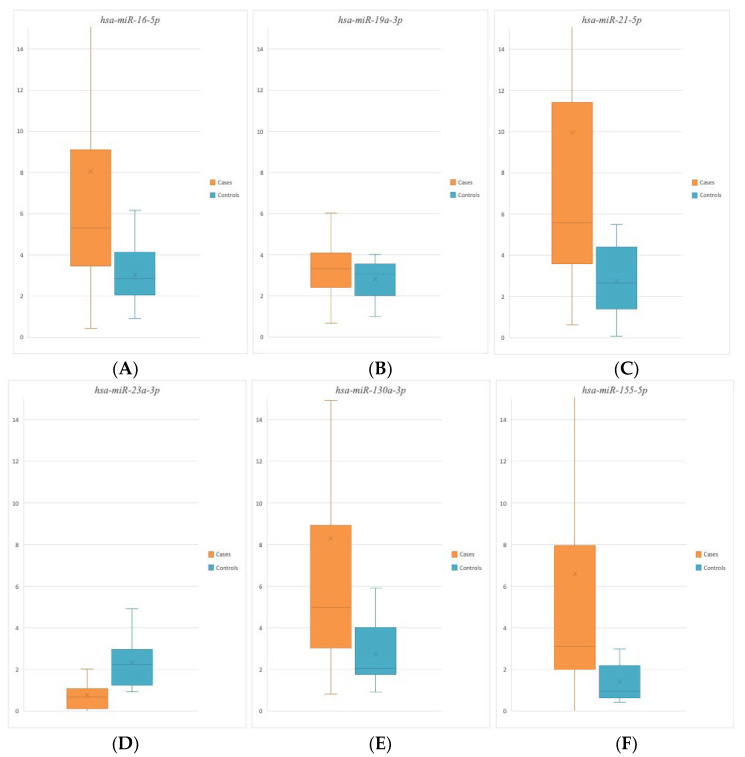
Box plot showing miRNA expression levels analysed in cases and controls: (**A**) hsa-miR-16-5p, (**B**) hsa-miR-19a-3p, (**C**) hsa-miR-21-5p, (**D**) hsa-miR-23a-3p, (**E**) hsa-miR-130a-3p, and (**F**) hsa-miR-155-5p.

In particular, overall higher expression levels were detected in cases compared to controls (upregulation) for miR-16-5p, miR-21-5p, miR-130a-3p, and miR-155-5p (Figure 3). On the other hand, lower expression levels were detected in cases compared to controls (downregulation) for miR-23a-3p (Figure 3).

In contrast, no significant variations in expression levels between cases and controls were detected for miR-19a-3p.

### 2.5. Subgroup Analysis: Correlation of Expression Profiles with Survival Time and Severity of Trauma

According to survival time, data analysis showed that miR-21-5p expression levels were approximately three times higher in the subgroup consisting of TBI with short survival (<1 day) than in the subgroups with no survival (fatal) and middle survival (1–7 days). In contrast, miR-21-5p expression levels were under-regulated in the subgroup of TBI with long survival (>7 days).

The analysis of miR-16-5p showed an approximately 6-fold upregulation in the short-survival subgroup (<1 day) and an approximately 4-fold upregulation in the middle-survival (1–7 days) and long-survival (>7 days) subgroups.

The expression levels of miR-130a were significantly higher in TBI cases with short (<1 day) and middle survival (1–7 days) than in TBI cases with no survival (fatal) or long survival (>7 days).

In contrast, there were no statistically significant differences in the expression levels of the remaining miRNAs investigated between the subgroups stratified by survival time.

**Figure 4:**
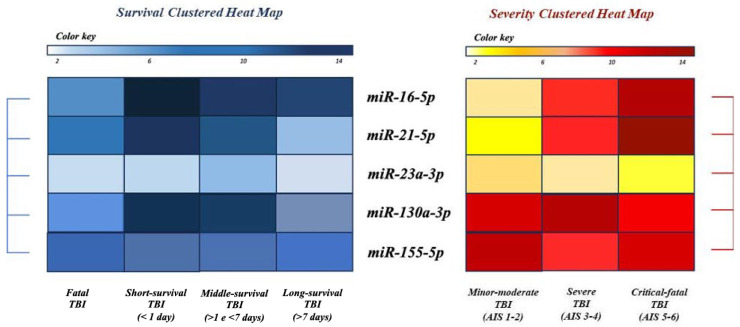
Clustered heat map showing the differential expression of the 5 miRNAs in the 3 subgroups defined on the basis of survival time (**left figure**) and on the basis of trauma severity (**right figure**). The coloured boxes represent the relative expression of the group (measured by Pearson’s distance metric), and the miRNAs are clustered in the heat map using a comprehensive clustering algorithm.

In relation to the severity of injury, data analysis showed a significantly higher expression of miR-16-5p and miR-21-5p in the critical–fatal TBI subgroup (AIS 5-6) than in the mild-to-moderate TBI (AIS 1-2) and severe TBI (AIS 3-4) subgroups. In contrast, there was no statistically significant variation in the expression levels of the remaining miRNAs investigated between the subgroups stratified by TBI severity (Figure 4).

## 3. Discussion

The mechanisms of injury described after TBI include neuroinflammation, synaptic dysfunction, protein aggregation, oxidative stress, blood–brain barrier damage, cerebral oedema, and cell death [42]. There are several cell populations involved in the pathophysiology of TBI, including astrocytes, microglia, oligodendrocytes, and endothelial cells, with inflammatory and reactive responses that may contribute to brain damage [43,44]. After TBI, cells of the innate immune system, such as microglia, can rapidly respond to the traumatic event by releasing cytokines and other pro-inflammatory molecules that contribute to the inflammatory response and local brain inflammation [45,46,47]. Cells of the adaptive immune system, such as T lymphocytes and B lymphocytes, may also be involved in the response to TBI through a more specific response that can be activated after significant tissue damage [48,49].

miRNAs represent potential and promising biomarkers of TBI, and several studies have evaluated their expressivity and demonstrated their dysregulation in relation to TBI, suggesting their involvement in pathophysiological mechanisms and their possible use in defining the diagnosis and prognosis of TBI [50,51].

miRNAs act through the mechanism of gene expression regulation by binding to specific target genes, called “target mRNAs”, and regulating their expression at the transcriptional or translational level [52,53]. This affects signalling pathways and cellular processes involving specific target genes regulated by different miRNAs.

The present study identified a panel of miRNAs that may be related to the mechanisms of damage in different brain cell populations, as well as those associated with neuroprotective and neurodegenerative mechanisms. The expression of these miRNAs was evaluated in brain tissue sections taken from subjects deceased as a result of TBI. This was compared with the expression of the same miRNAs in brain tissue sections taken from a control group of subjects deceased as a result of natural causes of cardiovascular origin.

Specifically, the following miRNAs were selected: hsa-miR-16-5p, hsa-miR-19a-3p, hsa-miR-21-5p, hsa-miR-23a-3p, hsa-miR-130a-3p, and hsa-miR-155-5p.

The described role of miR-16 [42,43,54,55] in relation to TBI is complex, depending on the specific context and conditions studied. miR-16 influences specific signalling pathways involved in traumatic brain damage and has been particularly linked to the regulation of several target genes involved in cell proliferation and glial apoptosis, including CDK6 (Cyclin-Dependent Kinase) and BCL-2 (B-cell lymphoma 2). Specifically, miR-16-5p overexpression can induce apoptosis by directly regulating BCL-2, an anti-apoptotic protein that prolongs cell survival by neutralising pro-apoptotic factors. The aberrant downregulation of BCL-2 disrupts mitochondrial membrane integrity, induces the mitochondrial release of pro-apoptotic proteins (e.g., cytochrome C and AIF), and triggers caspase activation and cytoskeletal degradation, leading to apoptosis. Studies conducted on glioma cell lines have shown that miR-16-5p binds directly to the 3′UTR of BCL-2 mRNA and deregulates cellular levels of BCL-2 mRNA and protein. These studies support an inhibitory role of miR-16-5p on glial cell proliferation, as well as its stimulating effects on apoptosis and increased caspase activity. MiR-16 has also been studied in relation to other brain disorders, such as Alzheimer’s disease, which may share some pathological features with TBI. It has been linked to the deposition of β-amyloid (Aβ), whose neurotoxicity is known to be a major cause of neurodegeneration. Additionally, miR-16 has been found to be significantly dysregulated in TBI patients, with significantly higher expression levels, particularly within the first 24 h post-trauma.

MiR-21 [38,46,47,48], one of the most studied miRNAs, is known to be involved in various cellular processes, including inflammation, apoptosis, and cell proliferation, and plays an important role in various pathological conditions, including traumatic brain injuries. In particular, in TBI, miR-21 expression is involved in blood–brain barrier damage, apoptosis, cell proliferation and differentiation, autophagy processes, and oxidative stress. Studies have shown that miR-21 acts at the astrocyte population level (reducing their activation), at the macrophage level (with pro-inflammatory effects by acting on the STAT3 signalling pathway, a protein involved in regulating the inflammatory response and cell activation), on dendritic cells, and on the T-cell line. In particular, miR-21 has been implicated in the regulation of the PTEN/PI3K/AKT pathway, affecting the expression of PTEN, which acts as an inhibitor of the PI3K/AKT pathway involved in cell survival and proliferation. MiR-21 can also influence the regulation of genes and proteins involved in blood–brain barrier permeability, indirectly affecting the inflammatory response and repair capacity. Recent studies have demonstrated high levels of miR-21 expression after TBI, and treatment with antago-miR-21 has been proposed as a potential therapy to reduce blood–brain barrier (BBB) damage [56,57]. A particular over-regulation of miR-21 has also been observed in the serum of patients with severe TBI (sTBI) compared to those with mild TBI (mTBI), at very early times and up to 15 days after the trauma. Furthermore, no increase was found in patients with musculoskeletal injuries without TBI, which is why miR-21 has been considered a potential new biomarker for TBI and a future therapeutic target [58].

MiR-23a [49] has been found to play a suppressive role in neuronal apoptosis and the inflammatory response in murine models of TBI. PTEN has been identified as a potential target gene of miR-23a. Specifically, studies have shown that miR-23a negatively regulates the expression levels of PTEN mRNA in primary cortical neurons, suppressing its expression both in vitro and in vivo. Since PTEN is a negative regulator of the AKT/mTOR pathway, it has been hypothesized that miR-23a plays a protective role in TBI by acting on the PTEN/AKT/mTOR pathway. Studies have shown reduced expression levels of miR-23a in TBI cases compared to controls, and the potential role of agomiR-23a as a therapeutic target has been evaluated.

MiR-130a [50,51], a micro-RNA involved in cellular and pathological processes, appears to play an important role in the context of traumatic brain injury (TBI). Recent studies have suggested that miR-130a may influence various aspects of the brain response to traumatic damage. Some of the target genes of this miRNA in the context of TBI include those involved in the regulation of inflammation, such as tumour necrosis factor alpha (TNF-α) and interleukin-6 (IL-6), as well as genes that regulate cell proliferation and cell death. miR-130a can influence the gene expression of PTEN, Homeobox Hox-A5, and AQP4 (aquaporin 4), leading to apoptosis, BBB damage, and brain oedema. A study conducted to investigate the role of miR-130a in BBB damage found an increase in its expression levels in microvascular endothelial cells and a corresponding increase in BBB permeability and brain oedema. It has been shown that the use of Antagomir-130a, an antagonist of miR-130a, could attenuate brain oedema, reduce BBB permeability, reduce brain lesion volume, and improve neurological function.

MiR-155 [52,53] has been implicated in the regulation of macrophage signalling mediated by Toll-like receptors (TLRs) 3 and 4, as well as interferon (IFN)-γ. This miRNA plays a key role in the modulation of neurotoxicity, macrophage and microglial activation, and in the regulation of the release of inflammatory mediators such as nitric oxide, cytokines, interleukins, and chemokine signalling. Recent studies have shown that experimentally induced traumatic brain injury (TBI) increases miR-155 expression in the damaged cortex and hippocampus [59,60]. Additionally, these injuries induce neuroinflammatory responses mediated by microglial cells, which are associated with neuronal loss and persistent neurological deficits [61,62]. Given the documented pro-inflammatory role of miR-155 in neurodegenerative diseases, its alteration in microglia induced by TBI may contribute to the chronicity of neuroinflammation and related neurodegeneration.

The data from the analyses performed in this study showed significantly different expression levels of miR-16-5p, miR-21-5p, miR-23a-3p, miR-130a-3p, and miR-155-5p between cases and controls (Figure 3). Conversely, no statistically significant differences were found between the study group and the control group for miR-19a-3p (Figure 3).

**Figure 5:**
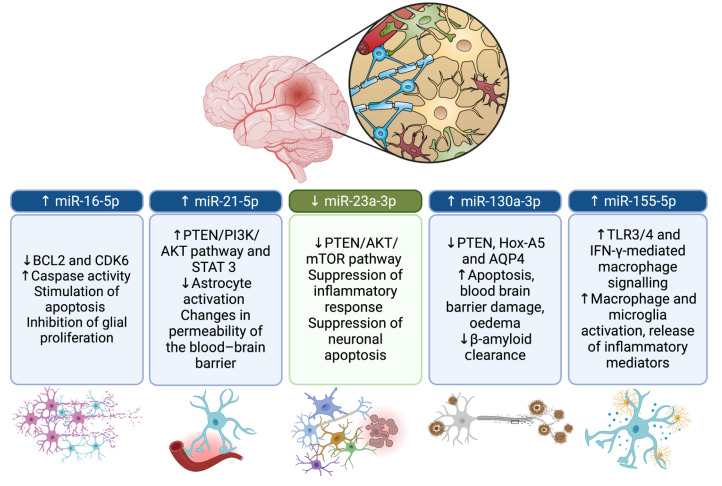
Expressivity (up- and downregulation) of the miRNAs examined in this study and their correlation with pathways, pathophysiological effects, and target cell populations.

Therefore, according to the analysis of the data obtained in the present study, it can be argued that the upregulation of miR-16-5p, miR-21-5p, miR-130a-3p, and miR-155-5p confirms the role of these miRNAs with pro-inflammatory, pro-apoptotic, and regulatory effects in signalling pathways involved in cell permeability and blood–brain barrier damage (Figure 5).

The downregulation of miR-23a-3p was consistent with the literature studies highlighting a potential protective role of this miRNA in TBI, confirming its suppressive effect on neuronal cell apoptosis and the inflammatory response (Figure 5).

Regarding the survival time associated with TBI, the significantly higher expression levels of miR-21-5p in the short-survival subgroup compared to the fatal TBI and middle-survival subgroups suggest that miR-21-5p may be associated with a rapid and specific response to severe injury. The approximately six-fold increase in miR-16-5p expression levels in the short-survival group and the approximately four-fold increase in the middle- and long-survival subgroups may indicate a key role of miR-16-5p in the response to TBI and short-term survival. Finally, the significant increase in miR-130a expression levels in TBI cases with short and middle survival suggests that miR-130a may be involved in the initial pathophysiological response following trauma.

In relation to trauma severity, the significant increase in miR-16-5p and miR-21-5p expression levels in the fatal–critical TBI subgroups (AIS 5-6) compared to the mild–moderate TBI (AIS 1-2) and severe TBI (AIS 3-4) subgroups suggests that these miRNAs may be involved in specific responses to severe injuries and trauma severity.

Overall, the results obtained from the data analysis suggest that miR-21-5p, miR-16-5p, and miR-130a may play an important role in the response to TBI and in determining the survival time and trauma severity in TBI patients.

## 4. Materials and Methods

### 4.1. Selection of Study Population

This multicentre collaborative study was carried out by the University of Salerno, the Sapienza University of Rome, and the University of Foggia.

The study population was selected from the cases that underwent autopsies on the orders of the Judicial Authority at the Forensic Medicine Departments, in a period between 2019 and 2023. In particular, in all the selected autopsy cases, tissue samples were taken and subsequently fixed in formalin and embedded in paraffin (FFPE) for subsequent histo-pathological examination.

A study population consisting of 40 cases of subjects with TBI-related deaths (study group) and 10 cases of subjects deceased suddenly from non-traumatic causes (control group) was then selected.

The study and control groups were selected based on specific inclusion and exclusion criteria (Table 3).

### 4.2. Definition of Subgroups

The study group (40 cases) was selected, and 4 subgroups were subsequently defined. Each subgroup consisted of cases distributed on the basis of survival time and divided into fatal TBI, TBI with short survival, TBI with medium survival, and TBI with long survival (Table 4).

The study group (40 cases) was stratified in parallel according to trauma severity. The definition of trauma severity was based on criteria derived from the Abbreviated Injury Scale (AIS) score [63,64], which assesses documented clinical parameters and injuries sustained at the time of trauma (Table 5).

The study group was further characterised on the basis of a number of parameters derived from medical records and/or autopsy and/or histo-pathological findings. For each case, the following parameters were taken into account: age, sex, cause of death, traumatic mechanism of death, type of brain injury found, postmortem interval (PMI), and whether surgical treatment was performed.

### 4.3. Sample Collection

For each case, a sample of brain tissue taken at autopsy was examined, fixed in formalin, and then embedded in paraffin (FFPE).

A total of 50 formalin-fixed, paraffin-embedded (FFPE) brain tissue samples were then processed, of which 40 belonged to the study group (corresponding for each case to the area of brain tissue most affected by the traumatic injury, selected on the basis of the findings of both macroscopic and microscopic autopsy examinations performed in all cases selected for this study) and 10 belonged to the control group.

### 4.4. miRNA Selection

The selection of miRNAs was based on a thorough literature review, which identified a panel of miRNAs potentially related to damage mechanisms in different brain cell populations, as well as to mechanisms of neuroprotection and neurodegeneration (Table 6).

Consequently, the following miRNAs were selected: hsa-miR-16-5p, hsa-miR-19a-3p, hsa-miR-21-5p, hsa-miR-23a-3p, hsa-miR-130a-3p, and hsa-miR-155-5p.

### 4.5. Total RNA Extraction

Four 10 μm thick sections were obtained from each formalin-fixed, paraffin-embedded (FFPE) tissue sample, and total RNA was isolated using the miRNeasy FFPE kit (Qiagen, Hilden, Germany), according to the manufacturer’s instructions.

Briefly, the protocol included an initial deparaffinization phase in which 1 mL of xylene and then 1 mL of ethanol were added to the tissue sections. The sample was digested with 240 μL PKD buffer and 10 μL of Proteinase K, incubated at 56 °C for 15 min and then at 80 °C for 15 min. It was then treated with DNase Booster Buffer in an amount equal to one-tenth of the total sample volume (approximately 16 μL) and with 10 μL of DNase stock solution and incubated at room temperature for 15 min. The sample was then treated with 320 μL RBC buffer, and 1120 μL of ethanol was added to the total lysate. Finally, the entire sample was transferred in two steps through the RNeasy MinElute spin column and centrifuged for 15 s at ≥8000× *g* (≥10,000 rpm). Total RNA was eluted in 17 μL of nuclease-free water. The RNA concentration was measured using a Nanodrop spectrophotometer with 1 μL of isolated RNA. Samples were stored at −80 °C.

### 4.6. Reverse Transcription and Real-Time PCR Assay of miRNA

For miRNA detection, 10 ng of total RNA, assessed using NanoDrop spectrophotometer (NanoDrop Technologies Inc., Wilmington, DE, USA), was processed using cDNA Synthesis Kit for reverse transcription (RT) of the miRNAs.

Real-time PCR (RT-qPCR) was performed for each miRNA (Table 7), using Taq DNA Polymerase and miRNA Assays Kits. Reverse-transcribed cDNA was diluted at a 1:10 ratio, and a total reaction volume of 20 µL was used and incubated at 95 °C for 20 seconds, 95 °C for 3 s (for 40 cycles), and 60 °C for 30 s (for 40 cycles). Each reaction was performed in duplicate.

For relative quantification, the 2-∆Ct method was applied, using the geometric means of miRNA hsa-miR-92a-3p as housekeeping.

### 4.7. Statistical Analysis

To achieve reproducibility and statistical significance, analyses were performed in duplicate for cases and controls. For normally distributed variables, a *t*-test or ANOVA was used. The association of the relationship between the miRNAs studied and the categorical variables was analysed using the Kruskal–Wallis test. A *p*-value of *p* < 0.05 was considered statistically significant. The statistical programme IBM SPSS STATISTICS was used for data analysis.

## 5. Conclusions

The results of this study provide significant evidence for the potential of the miRNAs studied as highly predictive biomarkers in distinguishing TBI cases from controls. These miRNAs could open up new perspectives for the postmortem diagnosis of TBI, offering the possibility of determining survival and severity.

Furthermore, the differential expression of the miRNAs studied according to trauma severity and survival time suggests that these miRNAs may also be valuable therapeutic targets. Indeed, since miRNAs are crucial regulators of many biological processes, including inflammation, apoptosis, and tissue repair, they could be modulated to develop new therapeutic strategies. In this sense, several studies in mouse models have already highlighted the potential role of miRNAs as therapeutic targets in TBI. However, the translation of basic research findings into clinical practice will require further studies, particularly to better understand the safety and efficacy of miRNA modulation in vivo.

In conclusion, the significant predictive power of these miRNAs could revolutionise the approach to the postmortem diagnosis of TBI, greatly improving the ability to identify and assess such injuries. These results suggest that miRNA analysis could become an essential tool in forensic investigations in the future, providing more accurate and detailed information about the nature and extent of TBI. The predictive power in relation to the timing and severity of trauma could ultimately contribute to the definition of the circumstances of death.

## Figures and Tables

**Table 1:** Study group characteristics.

Case	Sex	Age	Cause of Death	Injury Mechanism	Brain Injury	PMI	Severity	Survival	Surgery
**1**	M	57	Major blunt-fracture trauma	Road traffic accident	SDH, SAH, and cerebral and cerebellar intraparenchymal haemorrhage	74 h	AIS: 5 GOSE: 2 GCS: 3	1 day	No
**2**	M	19	Major blunt-fracture trauma	Road traffic accident	SAH, cortical haemorrhages, and pontine haemorrhages of Duret	60 h	AIS: 5 GOSE: 2 GCS: 3	3:30 h	Yes
**3**	M	64	Cardiorespiratory failure due to intracranial hypertension	Fall from standing	SDH acute over chronic	106 h	AIS: 1 GOSE: 7 GCS: 15	16 days	Yes
**4**	M	18	Major blunt-fracture trauma	Train accident	Temporoparietal skull fracture and SAH	52 h	AIS: 6 GOSE: 1 GCS: 3	0	No
**5**	M	21	Major blunt-fracture trauma	Road traffic accident	Anterior and middle skull base fractures, SAH, and cortical haemorrhages	57 h	AIS: 6 GOSE: 1 GCS: 3	0	No
**6**	F	65	MODS	Road traffic accident	SDH, SAH, and cerebral laceration–contusive focus	123 h	AIS: 2 GOSE: 4 GCS: 11	1.5 year	No
**7**	M	44	Major blunt-fracture trauma	Fall from a height	Cerebral and cerebellar SAH and intraparenchymal haemorrhage	108 h	AIS: 5 GOSE: 2 GCS: 3	~2–3 h	No
**8**	M	49	Major blunt-fracture trauma	Train accident	SAH	94 h	AIS: 6 GOSE: 1 GCS: 3	0	No
**9**	M	17	Major blunt-fracture trauma	Road traffic accident	Frontal, sphenoid, and maxillary skull fractures with haemosinus and SAH	98 h	AIS: 6 GOSE: 1 GCS: 3	0	No
**10**	M	63	Major blunt-fracture trauma	Road traffic accident	SAH	54 h	AIS: 3 GOSE: 3 GCS: 12	~2–3 h	No
**11**	M	65	Closed head trauma	Road traffic accident	Closed cranial vault fracture, SDH, SAH, and intraparenchymal haemorrhage	102 h	AIS: 5 GOSE: 2 GCS: 3	~2 days	No
**12**	M	62	MODS	Road traffic accident	SDH	75 h	AIS: 2 GOSE: 4 GCS: 15	51 days	No
**13**	M	60	Major blunt-fracture trauma	Road traffic accident	SAH and intraparenchymal cerebral haemorrhages	105 h	AIS: 3 GOSE: 4 GCS: 11	~4 days	No
**14**	M	12	Closed head trauma	Fall from standing	Closed head trauma of the posterior cranial fossa with intraparenchymal haemorrhage and cerebellar SAH	68 h	AIS: 6 GOSE: 1 GCS: 3	0	No
**15**	M	34	Major blunt-fracture trauma	Road traffic accident	Closed head trauma with fractures of the vault and skull base, SDH, cerebral and cerebellar SAH, and cerebral contusions	77 h	AIS: 6 GOSE: 1 GCS: 3	0	No
**16**	F	20	Major blunt-fracture trauma	Road traffic accident	SAH and brain contusions	54 h	AIS: 6 GOSE: 1 GCS: 3	0	No
**17**	M	14	Major blunt-fracture trauma	Road traffic accident	Splanchnocranium and skull base fractures and SAH	80 h	AIS: 6 GOSE: 1 GCS: 3	0	No
**18**	M	29	Major blunt-fracture trauma	Road traffic accident	Maxillary sinus fracture with haemosinus and SAH	74 h	AIS: 5 GOSE: 3 GCS: 3	~9:30 h	No
**19**	M	49	Major blunt-fracture trauma	Road traffic accident	Closed posterior cranial fossa fracture, SDH, SAH, and cerebral and cerebellar lacerated–contused foci	77 h	AIS: 3 GOSE: 6 GCS: 8	~2 days	Yes
**20**	M	60	Closed head trauma	Road traffic accident	Fractures of the splanchnocranium and skull base and SDH	53 h	AIS: 3 GOSE: 5 GCS: 9	~2 days	No
**21**	F	59	Major blunt-fracture trauma	Road traffic accident	Neurocranium fractures, SDH, SAH, and laceration–contusive focus	115 h	AIS: 3 GOSE: 4 GCS: 13	~7 days	Yes
**22**	M	20	Major blunt-fracture trauma	Train accident	Splanchnocranium and neurocranium fractures, SDH, SAH, and cerebral and cerebellar parenchymal lacerations	67 h	AIS: 6 GOSE: 1 GCS: 3	0	No
**23**	M	37	MODS	Road traffic accident	Splanchnocranium and skull base fractures, subdural blood layer, and subarachnoid spread	92 h	AIS: 2 GOSE: 5 GCS: 14	~25 days	No
**24**	F	59	Cardiorespiratory failure due to intracranial hypertension	Road traffic accident	Occipital fracture, SDH, and SAH	102 h	AIS: 2 GOSE: 4 GCS: 14	~27 days	Yes
**25**	M	41	Closed head trauma	Blunt trauma	Splanchnocranium and neurocranium fractures, SDH, and cerebral and cerebellar SAH	73 h	AIS: 6 GOSE: 1 GCS: 3	0	No
**26**	M	62	Major blunt-fracture trauma	Fall from a height	SAH with tetrahaemoventricle	56 h	AIS: 6 GOSE: 1 GCS: 3	0	No
**27**	M	21	Major blunt-fracture trauma	Road traffic accident	Fractures of the splanchnocranium, vault, and skull base, SDH and SAH layers, and contusion–haemorrhagic foci	88 h	AIS: 4 GOSE: 3 GCS: 12	~5 days	No
**28**	M	62	Closed head trauma	Road traffic accident	Fractures of the vault and skull base, SDH, SAH, and intraparenchymal haemorrhages	75 h	AIS: 4 GOSE: 3 GCS: 11	~3 days	No
**29**	M	65	Cardiorespiratory failure due to intracranial hypertension	Fall from standing	SDH, SAH, and intraparenchymal contusive foci	98 h	AIS: 4 GOSE: 3 GCS: 10	~6 days	No
**30**	F	64	Cardiorespiratory failure due to intracranial hypertension	Fall from standing	SDH and SAH	85 h	AIS: 5 GOSE: 2 GCS: 4	~6 days	No
**31**	F	63	Major blunt-fracture trauma	Road traffic accident	Neurocranium fracture, EDH, SDH, and SAH	94 h	AIS: 5 GOSE: 2 GCS: 6	~14 days	No
**32**	M	58	Cardiorespiratory failure due to intracranial hypertension	Fall from standing	Cranial vault fracture and cerebral and cerebellar SDH and SAH	58 h	AIS: 6 GOSE: 2 GCS: 3	~1:30 h	No
**33**	M	42	Cardiorespiratory failure due to intracranial hypertension from cerebral oedema	Fall from standing	Fractured floor of the orbit, flap of SDH, and SAH	63 h	AIS: 6 GOSE: 1 GCS: 3	~2 h	No
**34**	M	63	Major blunt-fracture trauma	Road traffic accident	Subacute SDH	94 h	AIS: 2 GOSE: 3 GCS: 15	~6 days	No
**35**	M	16	Major blunt-fracture trauma	Fall from a height	Cerebral and cerebellar SDH and SAH	62 h	AIS: 5 GOSE: 2 GCS: 3	~1 h	No
**36**	F	19	Major blunt-fracture trauma	Fall from a height	Splanchnocranium and neurocranium fractures and diffuse SAH	64 h	AIS: 6 GOSE: 1 GCS: 3	0	No
**37**	F	59	Cardiorespiratory failure due to intracranial hypertension	Road traffic accident	Cranial vault fracture, bilateral SDH, and SAH	112 h	AIS: 2 GOSE: 5 GCS: 14	~11 days	No
**38**	F	65	Cardiorespiratory failure due to intracranial hypertension	Fall from standing	SDH	74 h	AIS: 1 GOSE: 5 GCS: 15	~6:30 h	No
**39**	M	58	Cardiorespiratory failure due to intracranial hypertension	Road traffic accident	Neurocranium fractures, bilateral SDH, and bilateral SAH	78 h	AIS: 4 GOSE: 2 GCS: 3	~13 days	No
**40**	M	47	Cardiorespiratory failure due to intracranial hypertension	Road traffic accident	Neurocranium fractures, bilateral SDH, and bilateral SAH	70 h	AIS: 4 GOSE: 5 GCS: 15	~4 days	Yes

SDH: subdural haematoma. SAH: subarachnoid haemorrhage. AIS: Abbreviated Injury Scale. GOSE: Glasgow Outcome Scale—Extended. GCS: Glasgow Coma Scale. MODS: Multiple Organ Dysfunction Syndrome. ~: Indicates approximation.

**Table 2:** Control group characteristics.

Case	Sex	Age	Cause of Death
**1**	M	51	Sudden cardiac death (SCD)
**2**	M	62	Acute myocardial infarction (AMI)
**3**	F	65	Sudden cardiac death (SCD)
**4**	F	55	Sudden cardiac death (SCD)
**5**	F	59	Sudden cardiac death (SCD)
**6**	M	53	Arrhythmic-based acute heart failure secondary to arrhythmogenic cardiomyopathy (ARC)
**7**	F	60	Arrhythmic-based acute heart failure secondary to arrhythmogenic cardiomyopathy (ARC)
**8**	M	47	Sudden cardiac death (SCD)
**9**	M	24	Sudden cardiac death (SCD)
**10**	M	56	Acute pulmonary embolism (PE)

**Table 3:** Inclusion and exclusion criteria for the study population.

Inclusion Criteria
Study group: - Subjects aged 12–65 years; - Subjects deceased as a result of a traumatic event, for which a diagnosis of traumatic brain injury has been established.
Control group: - Subjects aged 12–65 years; - Subjects deceased as a result of natural causes with cardiovascular genesis.
**Exclusion Criteria**
- Subjects in childhood (<12 years old) or elderly subjects (>65 years old); - Subjects with a history and/or evidence of previous traumatic brain injury; - Subjects with pre-existing neurodegenerative diseases of the central nervous system and/or previous injuries and other brain diseases (neoplastic, ischaemic, haemorrhagic, infectious, inflammatory, autoimmune, and hereditary); - Subjects with acute and/or chronic drug intoxication; - Subjects on active drug therapy for the central and peripheral nervous system; - Subjects for whom anamnestic information and/or documentation of pre-existing pathologies could not be collected.

**Table 4:** Definition of subgroups based on survival time.

Fatal TBI	Head Injury without Survival (Fatal Traumatic Brain Injury, No Survival)
**Short-survival TBI**	Head injury with a survival time of less than 1 day (short survival < 1 day)
**Middle-survival TBI**	Head injury with a survival time of between 1 and 7 days (middle survival >1 and <7 days)
**Long-survival TBI**	Head injury with a survival time of more than 7 days (long survival > 7 days)

**Table 5:** Definition of subgroups based on severity of trauma.

**Minor–moderate TBI**	**AIS 1**: light brain injuries with headache, vertigo, no loss of consciousness, light cervical injuries, whiplash, abrasion, and contusion. **AIS 2**: concussion with or without skull fracture, less than 15 min unconsciousness, corneal tiny cracks, detachment of retina, and face or nose fracture without shifting.
**Severe TBI**	**AIS 3**: concussion with or without skull fracture, more than 15 min unconsciousness without severe neurological damages, closed and shifted or impressed skull fracture without unconsciousness or other injury indications in skull, loss of vision, shifted and/or open face bone fracture with antral or orbital implications, and cervical fracture without damage of spinal cord. **AIS 4**: closed and shifted or impressed skull fracture with severe neurological injuries.
**Critical–fatal TBI**	**AIS 5**: concussion with or without skull fracture with more than 12 h unconsciousness with haemorrhage in skull and/or critical neurological indications. **AIS 6**: death, partly or fully damaged brainstem or upper part of cervical area due to pressure or disruption. Fracture and/or wrench of upper part of cervical area with injuries of spinal cord.

**Table 6:** Characteristics of the miRNAs selected for this study.

miRNA	Protein/Pathway	Described Effects	References
**miR-16-5p**	BCL-2 CDK6	Glial proliferation and apoptosis Apoptosis and modulation of neuronal Ca^2+^ signalling β-Amyloid deposition	Krell et al., 2019 [65] Kim et al., 2019 [66]
**miR-19a-3p**	AQP4, Cx43 ADIPOR2	Oedema and expression of astrocytic aquaporin 4 Apoptosis Ischaemia/reperfusion damage	Julienne et al., 2018 [67] Vandebroek et al., 2020 [68]
**miR-21-5p**	PTEN PDCD4/PI3K/AKT/GSK-3β pathway STAT3	Blood–brain barrier damage Apoptosis, survival, proliferation and differentiation, and autophagy Oxidative stress and altered signalling	Pinchi et al., 2018 [38] Feng et al., 2018 [69] Xu et al., 2019 [70] Bai et al., 2022 [71]
**miR-23a-3p**	PTEN/AKT/mTOR pathway	Apoptosis	Li et al., 2020 [72]
**miR-130a-3p**	PTEN Homeobox Hox-A5 AQP4	Apoptosis Damage to the blood–brain barrier, oedema, expression of astrocytic aquaporin 4 M1, and clearance of β-amyloid by the glymphatic system	Wang et al., 2018 [73] Sepramaniam et al., 2012 [74]
**miR-155-5p**	Macrophage signalling mediated by Toll-like receptors (TLRs) 3 and 4 and interferon (IFN)-γ	Modulation of neurotoxicity Macrophage and microglia activation, modulation of the release of inflammatory mediators (nitric oxide, cytokines, interleukins), and chemokine signalling	Henry et al., 2019 [75] Li et al., 2017 [76]

**Table 7:** Micro-RNA sequences used in PCR.

miRNA Name	Accession Number	Primer Sequence
***hsa-miR-16-5p***	MIMAT0000069	UAGCAGCACGUAAAUAUUGGCG
***hsa-miR-19a-3p***	MIMAT0000073	UGUGCAAAUCUAUGCAAAACUGA
***hsa-miR-21-5p***	MIMAT0000076	UAGCUUAUCAGACUGAUGUUGA
***hsa-miR-23a-3p***	MIMAT0000078	AUCACAUUGCCAGGGAUUUCC
***hsa-miR-130a-3p***	MIMAT0000425	CAGUGCAAUGUUAAAAGGGCAU
***hsa-miR-155-5p***	MIMAT0000646	UUAAUGCUAAUCGUGAUAGGGGUU

## Data Availability

The data are included in legal cases in Italy, and for this reason, they are not available.
